# Dendritic Cells Reveal a Broad Range of MHC Class I Epitopes for HIV-1 in Persons with Suppressed Viral Load on Antiretroviral Therapy

**DOI:** 10.1371/journal.pone.0012936

**Published:** 2010-09-23

**Authors:** Xiao-Li Huang, Zheng Fan, LuAnn Borowski, Robbie B. Mailliard, Morgane Rolland, James I. Mullins, Richard D. Day, Charles R. Rinaldo

**Affiliations:** 1 Department of Infectious Diseases and Microbiology, Graduate School of Public Health and School of Medicine, University of Pittsburgh, Pittsburgh, Pennsylvania, United States of America; 2 Department of Microbiology, University of Washington, Seattle, Washington, United States of America; 3 Department of Biostatistics, Graduate School of Public Health and School of Medicine, University of Pittsburgh, Pittsburgh, Pennsylvania, United States of America; 4 Department of Pathology, Graduate School of Public Health and School of Medicine, University of Pittsburgh, Pittsburgh, Pennsylvania, United States of America; New York University, United States of America

## Abstract

**Background:**

HIV-1 remains sequestered during antiretroviral therapy (ART) and can resume high-level replication upon cessation of ART or development of drug resistance. Reactivity of memory CD8^+^ T lymphocytes to HIV-1 could potentially inhibit this residual viral replication, but is largely muted by ART in relation to suppression of viral antigen burden. Dendritic cells (DC) are important for MHC class I processing and presentation of peptide epitopes to memory CD8^+^ T cells, and could potentially be targeted to activate memory CD8^+^ T cells to a broad array of HIV-1 epitopes during ART.

**Principal Findings:**

We show for the first time that HIV-1 peptide-loaded, CD40L-matured DC from HIV-1 infected persons on ART induce IFN gamma production by CD8^+^ T cells specific for a much broader range and magnitude of Gag and Nef epitopes than do peptides without DC. The DC also reveal novel, MHC class I restricted, Gag and Nef epitopes that are able to induce polyfunctional T cells producing various combinations of IFN gamma, interleukin 2, tumor necrosis factor alpha, macrophage inhibitory protein 1 beta and the cytotoxic de-granulation molecule CD107a.

**Significance:**

There is an underlying, broad antigenic spectrum of anti-HIV-1, memory CD8^+^ T cell reactivity in persons on ART that is revealed by DC. This supports the use of DC-based immunotherapy for HIV-1 infection.

## Introduction

The breadth of CD8^+^ T cell reactivity specific for HIV-1 antigens is considered a key factor in host control of HIV-1 infection [1]. Production of interferon γ (IFNγ) by memory CD8^+^ T cells that are specific for a broad array of HIV-1 epitopes, especially those within the Gag protein, is associated with slower HIV-1 disease progression [2], [3]. Control of HIV-1 infection has also been linked to polyfunctional reactivity of memory CD8^+^ T cells, i.e., T cells that produce more than one immune mediator in response to HIV-1 antigens [4], particularly Gag [5], [6], [7], [8]. This has led to the concept that effective prophylactic and immunotherapeutic vaccines for HIV-1 will need to induce a broad, HIV-1 antigenic spectrum of CD8^+^ T cell reactivity.

Induction of broad and robust T cell reactivity could be particularly important in immunotherapy of HIV-1 infection during antiretroviral therapy (ART) [9]. However, virus-suppressive ART results in a contraction of anti-HIV-1, CD8^+^ memory T cell function related to the lower HIV-1 antigenic burden [10], [11], [12], [13]. Based on recent evidence that dendritic cells (DC) are important for activation of memory CD8^+^ T cell reactivity to influenza A virus, herpes simplex virus type 1 and human cytomegalovirus [14], [15], [16], [17], [18], we hypothesized that DC could enhance the breadth of T cell responses to HIV-1, particularly in persons on ART. In the present study, we therefore analyzed the breadth of memory, recall CD8^+^ T cell responses in vitro from HIV-1 infected subjects on ART to DC loaded with HIV-1 peptides. Our results show that HIV-1 peptide-loaded, mature DC induced IFNγ production to a much broader range of HIV-1 Gag and Nef epitopes than did peptides without DC. The MHC class I restricted Gag and Nef epitopes included novel ones that could activate polyfunctional T cells producing various combinations of IFNγ interleukin 2 (IL-2), TNFα, macrophage inhibitory protein 1β (MIP-1β) and the cytotoxic de-granulation molecule CD107a. This indicates that there is a broader and more robust array of memory CD8^+^ T cells specific for HIV-1 antigens circulating in persons on ART than has previously been appreciated, and supports use of DC-based immune therapies.

## Methods

### Study subjects

This research was part of the Pittsburgh Multicenter AIDS Cohort Study (MACS), an investigation of the natural history of HIV infection, and was approved by the University of Pittsburgh Institutional Review Board. 7 HIV-1 seropositive homosexual men on ART were randomly selected for study from the Pittsburgh, PA, portion of the MACS (Table S1). Four HIV-1 seronegative persons were included as controls. All study subjects gave written informed consent.

### DC cultures

To obtain immature DC, CD14^+^ monocytes were positively selected from peripheral blood mononuclear cells (PBMC) using anti-CD14 monoclonal antibody (mAb)-coated magnetic microbeads (StemCell Technologies, Vancouver, Canada) to a purity of >96%, cultured for 5 to 6 days in AIM V medium (GIBCO, Grand Island, NY) containing 1000 U/ml of recombinant IL-4 (R & D Systems, Minneapolis, Minn.) and 1000 U/ml of recombinant granulocyte-monocyte colony stimulating factor (GM-CSF) (Amgen, Seattle, WA). Fresh IL-4 and GM-CSF were added every other day. The DC were treated with maturation factor CD40L (0.5 µg/ml; Amgen or Alexis, San Diego, CA) for 40 h to induce DC maturation.

The number of viable DC was determined by typical morphology in trypan blue dye-stained preparations. The maturation status of the DC was determined by flow cytometry as the percent positive and mean fluorescent intensity of expression of MHC class II (HLA-DR), MHC class I (HLA ABC), CD80, CD86 and CD83. Viable DC displayed a characteristic DC morphology and cell surface marker expression and responded to stimulation with CD40L.

### Synthetic peptides

A library of HIV-1 peptides (consecutive 15mers overlapping by 11 amino acids) spanning the consensus B HIV-1 proteome was obtained through the AIDS Research and Reference Reagent Program, Division of AIDS, NIAID, NIH. These were used as singlets or in pools of consecutive peptides. Known, “A-list” epitopes were identified by the Los Alamos CTL/CD8^+^ T Cell Epitope Database [19]. Potential new epitopes were determined based on HLA anchor residue motifs within protein sequences for specified HLA alleles using the HLA Binding Motif Scanner, which is based on two motif libraries [20], [21]. HIV-1 peptides with various N and C terminal truncations and extensions were synthesized for determination of HLA association (SynBioSci, Livermore, CA). Preliminary dose-response experiments were done to determine the optimal concentration of peptides to be used in the T cell functional assays.

### ELISPOT assay

An ELISPOT assay modified from AIDS Clinical Trials Group protocol A5181 was used to determine single cell IFNγ production [22]. Briefly, plates were pre-labeled with coating antibody (1-D1K; 100 µL/well or 1 µg/mL solution; Mabtech, Stockholm, Sweden), incubated overnight at 4°C, washed 4 times with PBS and blocked with RPMI 1640 medium with 10% heat-inactivated FCS (RPMI-10% FCS) (Gemini Bio-Products, West Sacramento, CA) for 1 h at 37°C. After decanting the blocking medium, DC in RPMI-10% FCS were added to the wells and loaded with peptides (5–10 µg/ml per peptide) for 2 h at 37**°**C. Responder cells were autologous PBMC or CD8^+^ T cells (96–98% pure) positively selected from PBMC using anti-CD8 mAb-coated, magnetic microbeads (StemCell). The peptide-loaded DC (stimulators) were washed to remove excess peptide and mixed with responder cells at a responder-to-stimulator [R:S] cell ratio of 10 to 1 and incubated for 18 h with peptide-loaded DC at 37°C in a 5% CO_2_ atmosphere. The wells were washed with PBS and treated with biotinylated anti-IFN-γ mAb (1 µl/ml; 100 µl/well) and incubated at 37**°**C for 3 hours. Avidin-peroxidase (100 µl/well) was added after the biotinylated antibody was decanted and the plates were washed four times with PBS-0.05% Tween 20 (Fisher Scientific, Pittsburgh, PA). Diaminobenzidine solution (100 µL/well; Sigma, St. Louis, MO) was added to each well for 5 minutes at room temperature. The plates were washed and air-dried overnight. A negative control (medium without peptides), and 2 positive controls – CEF (1 µg/ml), which is a mixture of human cytomegalovirus, Epstein-Barr virus and influenza A virus [23] (NIH AIDS Research & Reference Reagent Program), and staphylococcus enterotoxin B (SEB, 0.5 µg/ml; Sigma, St. Louis, MO), were included in each assay. After the plates were processed for staining of IFNγ, the spots were counted with an ELISPOT reader system (Cell Technology, Columbia, MD). Data were expressed as spot-forming cells (SFC) per 10^6^ cells. The results were considered positive if the number of SFC in the peptide-stimulated cultures was more than 50 and above the mean plus two standard deviations of SFC in cultures with medium alone.

In some experiments, HLA restriction of the T cell responses was confirmed by ELISPOT assay using a panel of EBV-transformed B cell lines (BLCL) matched with the effector cells at only one MHC class I allele.

### Surface and intracellular staining (ICS)

Frozen-thawed PBMC were suspended to 2×10^6^/ml in RPMI-10% FCS and rested overnight at 37°C. The PBMC were then cultured with 2 µl each of T cell costimulatory mAb specific for CD28 and CD49d (αCD28/49d, 1 µg/ml; BD Biosciences), monensin (5 µg/ml; Sigma) and brefeldin A (5 µg/ml; Sigma) to inhibit extracellular release of the immune mediator CD107a-PECy5 (20 µl; BD PharMingen, San Diego, CA), and peptides or peptide pools (5 µg/ml). In some experiments, DC loaded with peptide (5 µg/ml) were used at a 1∶10 ratio with PBMC but without αCD28/49d. Negative controls (without peptides) and positive controls (CEF, 1 µg/ml and SEB, 1 µg/ml) were included in each assay. Cells were incubated for 6 h at 37°C and then kept at 4°C for 16 h. The cells were washed, fixed using the Cytofix/Cytoperm kit (BD PharMingen) and stained with mAb CD8-APC Cy7, CD4-APC Cy7, IL-2-APC (BD Biosciences), CD3-PE Cy7, IFNγ-FITC, MIP-1β-PE (BD PharMingen) and TNFα-PB (eBiosciences). Following staining, the cells were washed, fixed and analyzed with an LSR II flow cytometer (BD Immunocytometry Systems), with 200,000 to 1,000,000 events collected per sample. T lymphocyte subsets were analyzed by first identifying and gating the whole lymphocyte population according to light scatter properties (FSC and SSC), followed by gating T cell subsets based on the expression of the surface markers CD3 and CD8, as well as the intracellular expression of IL-2, IFNγ, TNFα, MIP-1β and CD107a compared to negative controls. All data were background-subtracted using the non-antigen stimulated control and analyzed by FlowJo (version 7.2.5; TreeStar, Ashland, OR) and SPICE (version 4.1.6). The expression of CD107a, IFNγ, IL-2, MIP-1β, TNFα and T cell surface markers was quantified separately and in combination.

### Statistical analysis

Statistical analyses were carried out to test three specific hypotheses: (a) PBMC and CD8^+^ T cell responses to HIV-1 peptides are stronger in the presence than in the absence of DC; (b) CD8^+^ T responses to HIV-1 peptides exceed PBMC responses both in the presence and in the absence of DC; and (c) relative changes in the level of response to HIV-1 peptides is highly correlated between CD8^+^ T cells and PBMC both in the presence and the absence of DC. The first two hypotheses were tested using the binomial sign test. In each case, the peptide data was transformed into a binary variable and tested against the null hypothesis (H_0_ = 0.5) using a one-sided alternative. For the first two hypotheses, we also calculated mean ratios and 95% confidence intervals in order to provide a better sense of the size of the comparative responses. For the third hypothesis, we ranked the size of the response observed to each of the HIV-1 peptides by PBMC and CD8^+^ T cells both in the presence and the absence of DC. Under both of the latter conditions (presence or absence of DC), we calculated Spearman rank order correlations between the PBMC and CD8^+^ T cell responses and the statistical significance (p-value) of each correlation. We used the Scheffe multiple comparison test and chi-square test for analysis of the polyfunctional T cell results.

## Results

### Enhanced breadth of IFNγ production by CD8^+^ T cells stimulated by DC loaded with peptide pools representing the HIV-1 proteome

**Figure 1:**
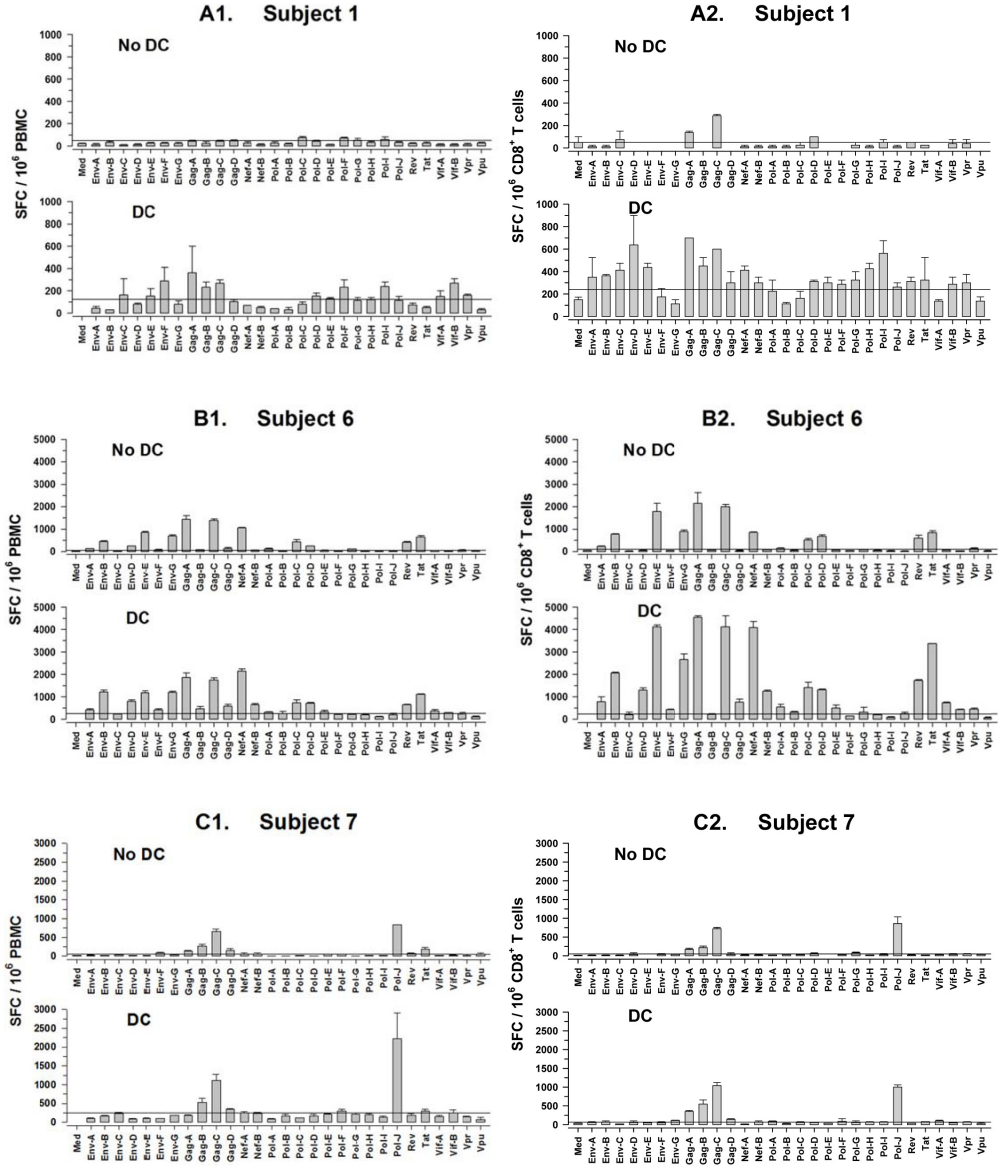
Single cell IFN gamma production by purified CD8^+^ T cells compared to PBMC in response to DC loaded with peptides spanning the HIV-1 proteome. A library of HIV-1 15mer peptides overlapping by 11 aa that spanned the HIV-1 proteome was arranged into 29 pools of 19–32 peptides per pool. HIV-1-specific reactivity in PBMC (A1, B1 and C1) and purified CD8^+^ T cells (A2, B2 and C2) was assessed in 3 HIV-1 infected persons (subjects 1, 6 and 7) in response to CD40L-matured DC that were loaded with each of the peptide pools. Mean + SE in triplicate cultures. The horizontal lines above the abscissa delineate the positive cutoff for each subject's T cell response.

We first examined the effects of DC loaded with a library of overlapping 15mer peptides spanning the HIV-1 proteome arranged into 29 pools of 19–32 peptides each on T cell reactivity in HIV-1 infected persons on ART. We have previously shown that CD40L-matured DC loaded with pools of ≤32 HIV-1 peptides are optimal for stimulation of CD8^+^ T cell responses [24]. In the present study we found that peptide-loaded, CD40L-treated DC induced higher levels of peptide-specific, IFNγ production across the HIV-1 proteome in PBMC compared to that stimulated by the HIV-1 peptide pools without DC (P<0.05) (Fig. 1 A1, B1, C1). No IFNγ responses were observed using peptide-stimulated PBMC or CD8^+^ T cells from 3 HIV-1 seronegative, uninfected persons, with or without DC (data not shown). Furthermore, using purified CD8^+^ T cells as responders, we confirmed that the broad reactivity induced by the peptide-loaded DC was mediated by CD8^+^ T cells, as there was a correlation between the total response of the purified CD8^+^ T cells and the PBMC to the HIV-1 peptide pools (r = 0.845, P<0.001) (Fig. 1 A2, B2, C2). This enhanced breadth was noted by a 6.6 mean ratio increase (CI 4.53-8.67, P<0.001) in the total CD8^+^ T cell positive response to peptides with DC compared to stimulation with peptides without DC. Finally, using peptide-loaded DC, we detected CD8^+^ T cell responses to 21/29, 22/29 and 7/29 peptide pools in subjects 1, 6 and 7, respectively. These included reactivity to peptides within each HIV-1 protein except Vpu for subjects 1 and 6, and to peptides within Env, Gag, Pol and Vif for subject 7. Finally, the overall magnitude of the IFNγ responses was greater in purified CD8^+^ T cells than in the PBMC, with a mean ratio of the CD8^+^ T cell response to the PBMC response of 1.20 (CI 0.98-1.42, P<0.001) for the peptides in the absence of DC, and 1.57 (CI 1.35-1.80, P<0.001) in the presence of DC.

These results indicate that the IFNγ production in response to DC loaded with HIV-1 15mer peptides was produced mainly by CD8^+^ T cells in the PBMC cultures. These immune responses were of significantly greater magnitude and breadth across the whole HIV-1 proteome (except Vpu) compared to those induced by conventional stimulation with peptides without DC in HIV-1 infected persons on ART.

### Enhanced breadth of T cell IFNγ production stimulated by DC loaded with single HIV-1 Nef 15-mer peptides

**Figure 2:**
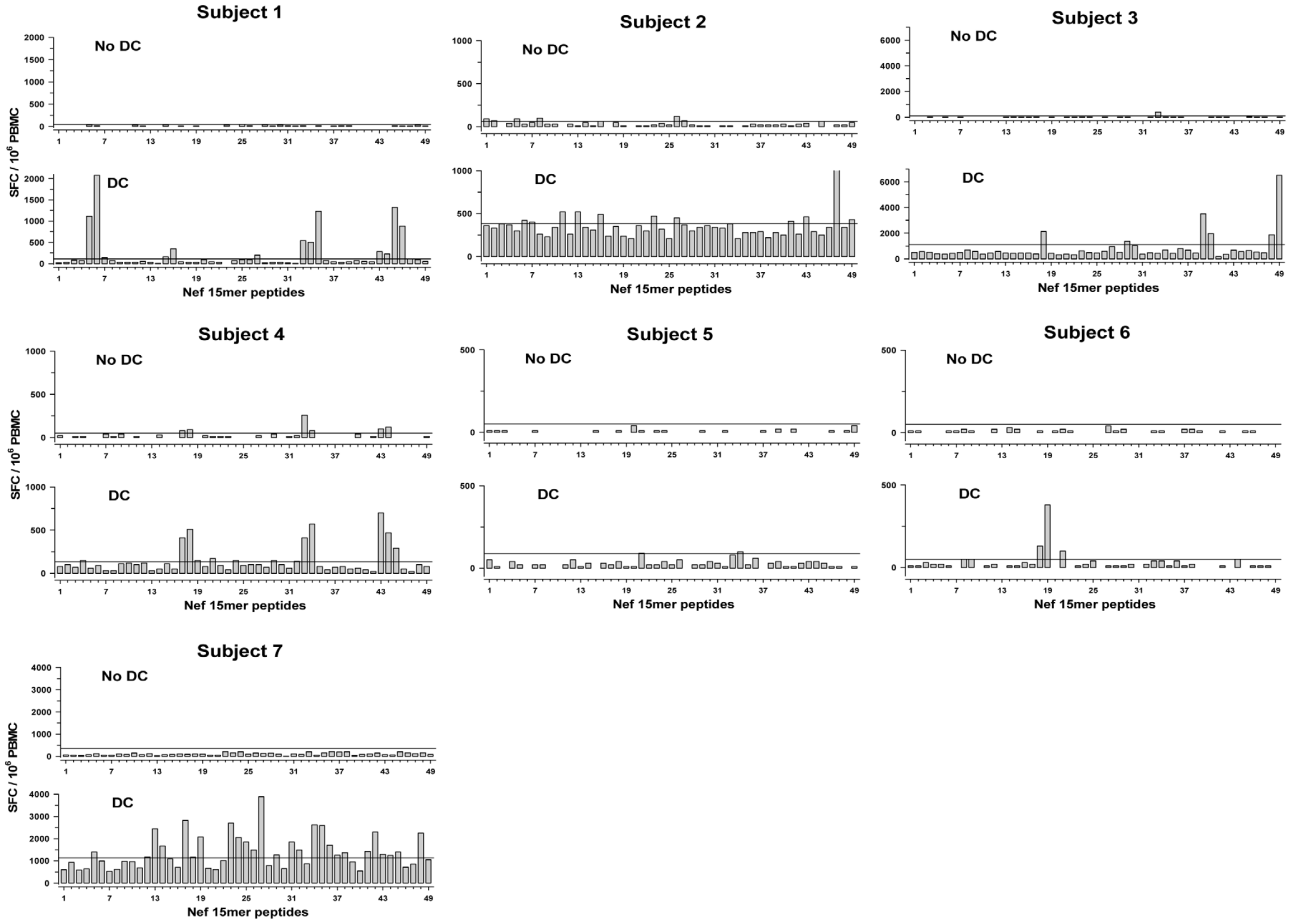
Enhanced single cell IFN gamma production stimulated by DC loaded with HIV-1 Nef 15mer peptides. CD40L-matured DC were loaded with single HIV-1 15-mer peptides spanning Nef and compared to peptides without DC for stimulation of HIV-1 specific IFN gamma production in 7 HIV-1 infected subjects on ART. The horizontal lines above the abscissa delineate the positive cutoff for each subject's T cell response.

We next focused on HIV-1 specific IFNγ production in response to CD40L-treated DC that were loaded with single peptides spanning 49 consecutive HIV-1 Nef 15mers overlapping by 11 amino acids in HIV-1 infected persons on ART. As expected, the number of positive responses to Nef varied among these genetically disparate study subjects (Fig. 2). Of the 343 possible T cell responses to the 49 Nef peptides among the 7 subjects, there were 74 (21.6%) positive T cell responses to DC loaded with the Nef peptides compared to only 5 (1.5%) positive responses to Nef peptides without DC (P<0.001). There were 7 (2%) common responses to peptides with and without DC. DC enhanced the number of Nef peptide-responding T cells by an average of 23.4 fold as compared to T cells stimulated directly with the peptides (no DC) (P<0.001 for unadjusted for background responses, p = 0.023 for adjusted values) (data not shown). Moreover, the positive responses were greater with DC loaded with Nef peptides than with Nef peptides without DC (P<0.001). The enhanced T cell responses to Nef were HIV-1 specific, reproducible in 2 subjects re-evaluated (P =  ns) and not associated with CD4^+^ T cell counts or viral load in the 7 HIV-1 infected subjects (data not shown).

Based on MHC class I alleles of the 7 subjects, we next determined the known and potential Nef epitopes associated with the T cell responses. There were 19 known Nef epitopes matched for the MHC class I alleles of the 7 subjects within the 42 Nef 15mers that induced positive responses (Table S2). Of these, the broadest IFN-γ responses to the Nef 15mer peptides were observed for subject 7 and the most restricted for subject 5 (Fig. 2). There were also responses to the 15mer Nef peptides that were not associated with a known epitope matched for the study subjects' MHC class I alleles for 24/42 (57%) of the reactive Nef peptides. Of the 37 total responses to these 24 peptides, 35 (94.6%) only occurred in response to peptide with DC, whereas 2 (5.4%) were in response to peptide with and without DC. There were also over 60 potential new MHC class I-restricted epitopes identified by their binding motifs within the positive Nef 15mer peptides (data not shown).

Taken together, these results show that DC from HIV-1 infected subjects on ART can process 15mer peptides for stimulation of responses against a broad range of known and potential Nef epitope in greater magnitude and breadth compared to that stimulated by these Nef peptides without DC.

### Novel HLA B*2703 Nef epitope revealed by stimulation with peptide-loaded DC

**Figure 3:**
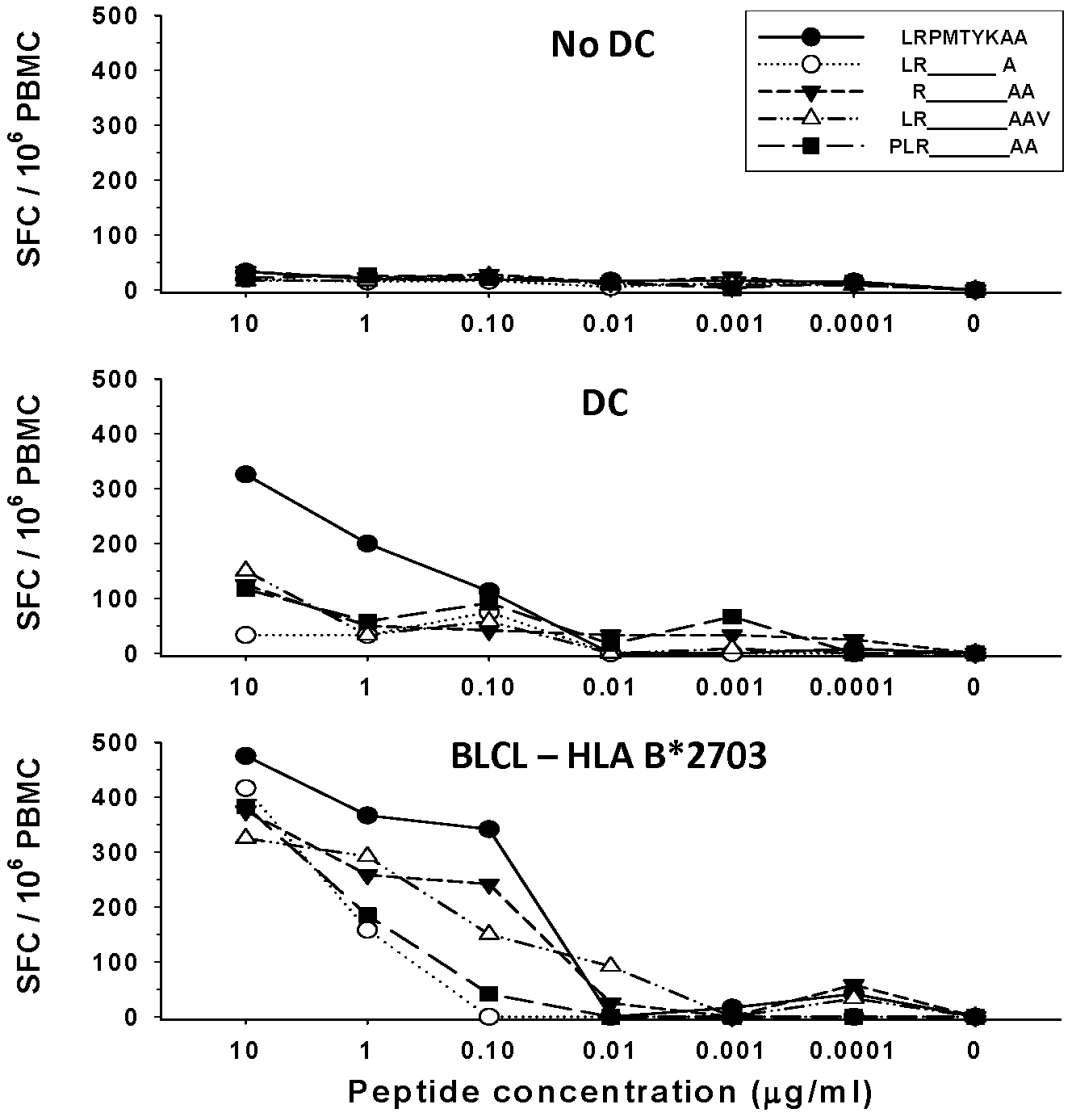
Stimulation of T cells with peptide-loaded DC reveals a novel HLA B*2703 restricted, Nef epitope determined by single cell IFN gamma production. Using PBMC from HIV-1 infected subject 6 and in silico predictions, a new HLA B*2703-restricted epitope Nef_76–84_ (LRPMTYKAA) was revealed within 15mer Nef_73–87_. The PBMC were stimulated with different concentrations of N and C terminal extensions and truncations of this peptide without DC (No DC), with DC (DC), and with BLCL matched at only one MHC class I allele (B*2703) to confirm the HLA restriction of the responses.

To verify recognition of novel MHC class I Nef epitopes revealed by DC, we mapped a minimal epitope within the 15mer Nef_73–87_ (QVPLRPMTYKAAVDL) peptide. We found that stimulation with peptide-loaded DC, but not with peptide without DC, resulted in positive IFN-γ responses in PBMC from 3 HIV-1 infected subjects who shared HLA B*2703 (peptide 19, Table S2 and subjects 4, 6 and 7, Fig. 2). We then focused on the internal 9mer LRPMTYKAA that was predicted to be restricted by HLA B*2703. Using cells derived from subject 6, DC loaded with Nef_76–84_ (LRPMTYKAA) stimulated the highest levels of IFNγ compared to N and C terminal extended and truncated peptides, in a concentration-dependent manner (Fig. 3, DC vs No DC). We confirmed the MHC class I restriction of this response using BLCL as APC that were MHC class I-matched only for HLA B*2703 (Fig. 3, BLCL-HLA B*2703).

**Figure 4:**
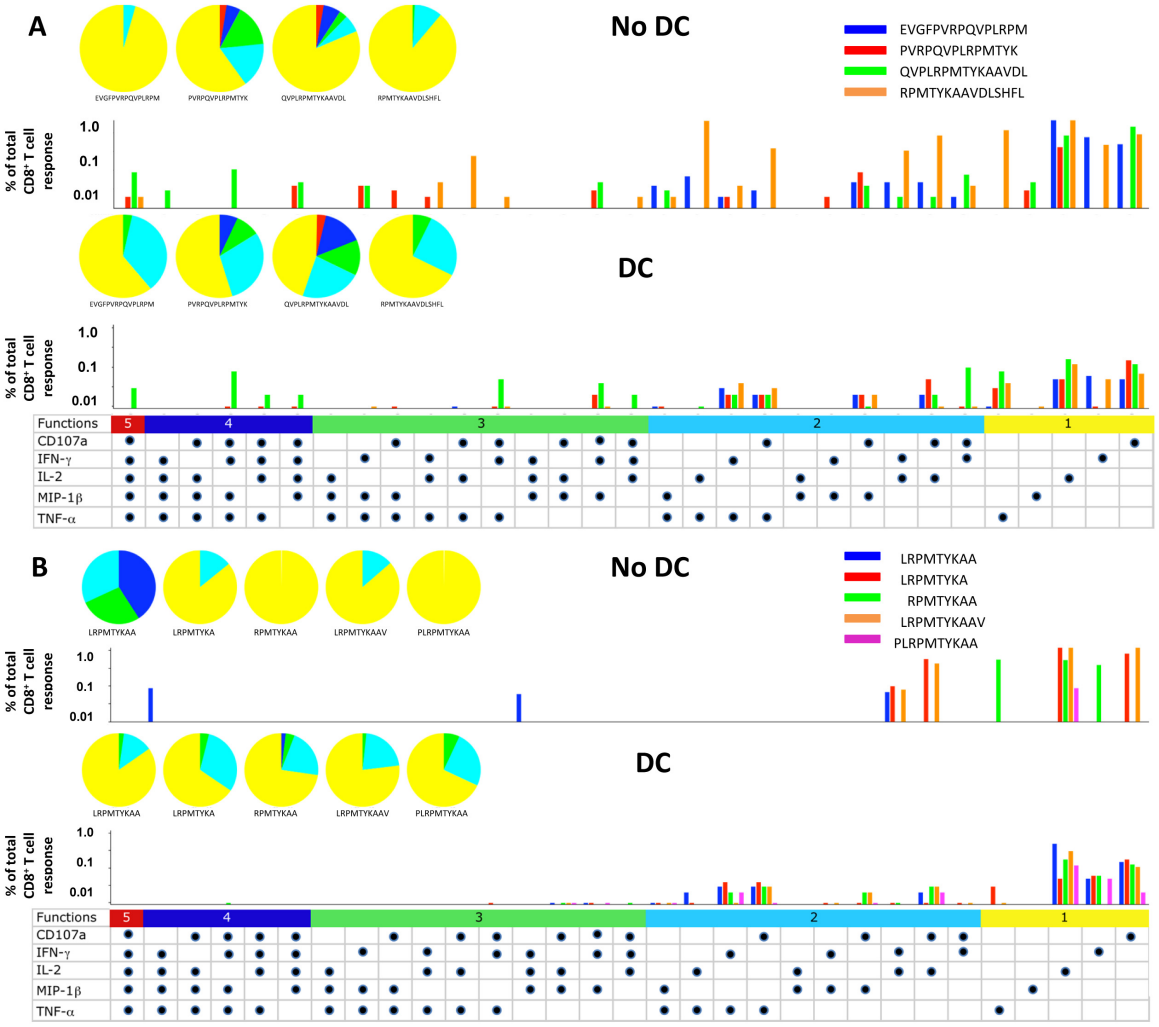
Induction of polyfunctional CD8^+^ T cells by DC loaded with a novel HLA B*2703 Nef epitope. Production of 5 immune mediators by CD8^+^ T cells from HIV-1 infected subject 6 was assessed in response to 4 overlapping 15mer peptides spanning Nef_65–91_(i.e., Nef_65–79_, Nef_69–83_, Nef_73–87_, and Nef_77–91_), with and without DC (Fig. 4A), and the in silico predicted optimal 9mer epitope Nef_76–84_ (LRPMTYKAA) and 4 N and C terminal variants with and without DC (Fig. 4B). The 5 color pie charts show the relative proportions of immune mediators produced in combinations of 1 to 5 per CD8^+^ T cell, and the color bar graphs represent the percentage of T cells responding to each individual 15mer (Fig. 4A) or 8-10mer peptides (Fig. 4B). The horizontal bars with different colors represent the percentage of CD8^+^ T cells producing one of the 5 immune mediators in response to the peptides. Each dot represents production of CD107a, IFN gamma, IL-2, MIP-1 beta or TNF alpha.

To assess the effects of DC on the breadth of the polyfunctional CD8^+^ T cell response, we studied Nef_73–87_ using multiparameter flow cytometry of PBMC stimulated with CD40L-matured DC loaded with peptide [24]. 3 of the 4 overlapping, 15mer peptides containing portions of the optimal epitope (Nef_76–84_) detected by the ELISPOT assay, i.e., Nef_69–83_, Nef_73–87_ and Nef_77–91_, induced the greatest polyfunctional CD8^+^ T cell responses when presented without DC (P<0.01 compared to N flanking Nef_65–79_) (Fig. 4A, no DC). This included CD8^+^ T cells producing all 5 of the immune mediators, i.e., CD107a, IFNγ, IL-2, MIP-1β and TNFα (P<0.01). With DC, the Nef_69–83_ and Nef_73–87_15mers induced the greatest polyfunctional CD8^+^ T cell responses (P<0.05 compared to Nef_65–79_ and Nef_77–91_) (Fig. 4A, DC). Among the 8-10mer peptides from this region presented without DC, the greatest polyfunctional response was induced by Nef_76–84_. This peptide without DC induced a trend towards a higher number of polyfunctional CD8^+^ T cells producing 2-to-4 immune mediators, i.e., IFNγ and IL-2, as well as MIP-1β and TNFα, compared to the 4 N and C terminal extended and truncated peptides (P = 0.07) (Fig. 4B, no DC). In contrast to stimulation without DC, there was no distinct polyfunctional CD8^+^ T cell response to DC loaded with each of the 5 8-10mer variants (P =  ns) (Fig. 4B, DC). Mostly monofunctional and dual polyfunctional T cells were stimulated by these peptides with DC.

These results show that DC revealed a new, HLA B*2703 epitope, Nef_76–84_, by a conventional ELISPOT in individuals on ART that was unrecognized using direct stimulation of PBMC with peptide alone. Monofunctional and polyfunctional ICS responses supported that these overlapping Nef 15mers contained a dominant CD8^+^ T cell epitope. However, a range of less definitive, CD8^+^ T cell activity was noted against Nef_76–84_ as well as to the N and C terminal variants, with and without DC.

### Enhanced breadth of T cell IFNγ production stimulated by DC loaded with single HIV-1 Gag 15-mer peptides

**Figure 5:**
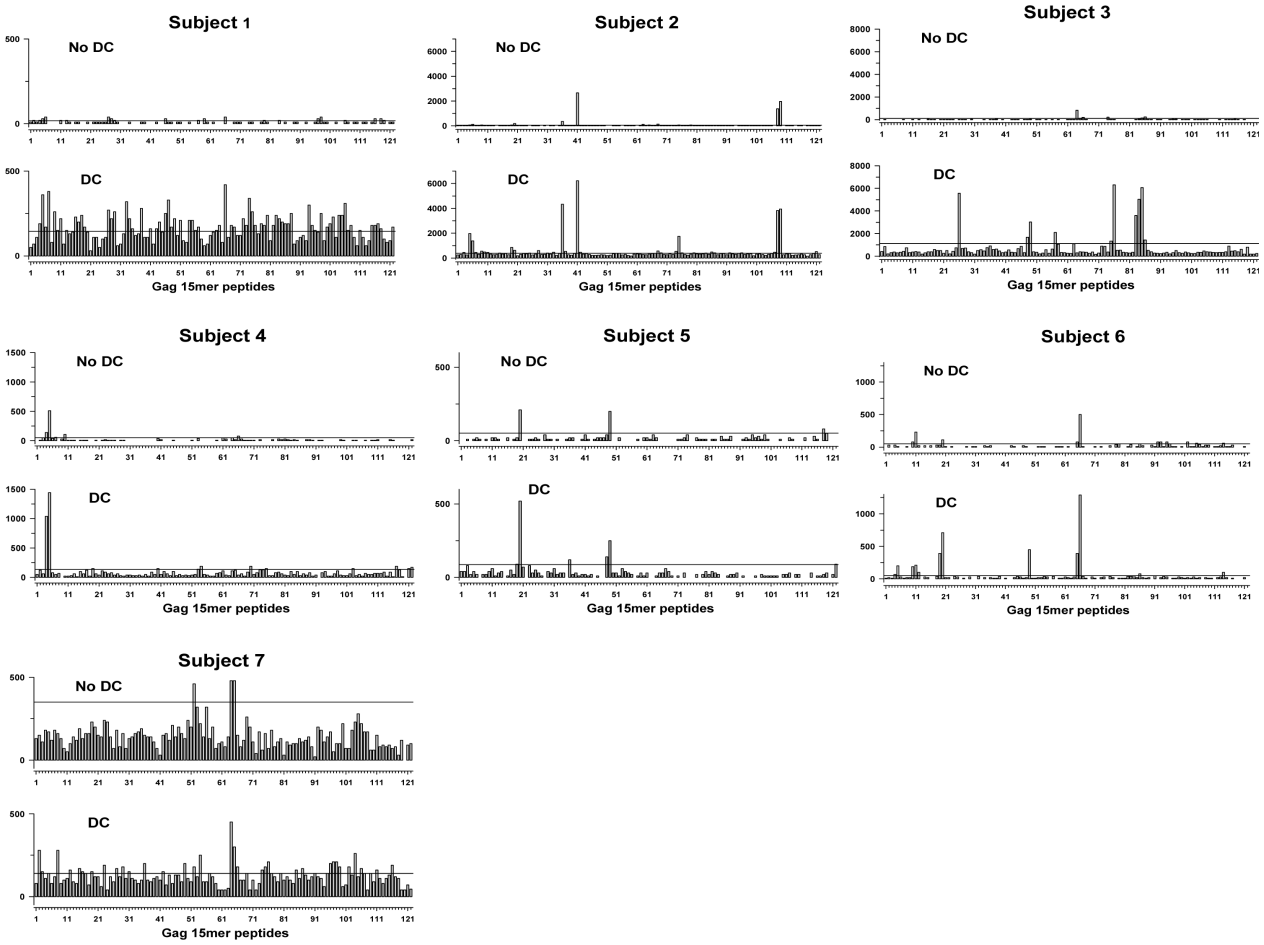
Enhanced single cell IFN gamma production stimulated by DC loaded with HIV-1 Gag 15mer peptides. CD40L-matured DC were loaded with single 15mer peptides spanning Gag p55 and compared to these peptides without DC for stimulation of HIV-1 specific, IFN gamma production in 7 HIV-1 infected subjects on ART. The horizontal lines above the abscissa delineate the positive cutoff for each subject's T cell response.

We next determined HIV-1 specific IFNγ production in response to CD40L-treated DC that were loaded with single peptides spanning HIV-1 Gag (122 consecutive 15mers overlapping by 11 amino acids) in HIV-1 infected persons on ART. Overall, there were responses to 80 of the 122 peptides by PBMC from the 7 subjects presented with or without DC (Fig. 5). Subjects 1, 2 and 7 had the broadest responses, i.e., 33, 35 and 33 responses, respectively. In contrast, only 7 peptides induced T cell responses from subject 5.

Of the total of 854 possible responses to the 122 Gag peptides among the 7 subjects, there were 114 (13.3%) responses to DC loaded with Gag peptides compared to only 14 (1.6%) responses to peptides without DC (P<0.001). There were 24 (2.8%) common responses to peptides with and without DC. Higher magnitude responses were observed to DC loaded with peptides compared to peptides without DC (P<0.001). DC enhanced the number of Gag peptide-responding T cells by an average of 8.5 fold (P<0.001).

Enhanced T cell responses to Gag were not associated with CD4^+^ T cell counts or viral load in the 7 infected subjects (data not shown). Repeat testing of different blood samples from 2 of these subjects resulted in IFNγ production comparable to the previous responses (P =  ns; data not shown). IFNγ production was HIV-1 immune specific, as there was little or no IFNγ production induced by the Gag peptides with or without DC in 4 HIV-1 negative controls (data not shown).

We assessed these data for known and potential new Gag epitopes based on MHC class I alleles of the 7 subjects. Results in Table S3 show that there were 57 known Gag epitopes matched for the MHC class I alleles of the 7 subjects within the 80 Gag 15mers that induced positive responses. Known MHC class I epitopes mostly clustered within Gag p17_5–31_, p17_73–103_, p24_5–27_, p24_57–95_, p24_117–179_ and p24_205–227_. The breadth of responses varied across subjects (Fig. 5; Table S3). 44 (55%) of the 80 reactive Gag peptides were not associated with a known epitope matched for the subjects' MHC class I alleles, corresponding to a total of 68 responses: 60 (88.2%) were in response to peptide with DC, 5 (7.4%) were in response to peptide alone, and 3 (4.4%) were in response to both. Based on prediction models for peptide binding to the various MHC class I alleles of these 7 subjects, there were over 200 potential new Gag epitopes identified within the 80 positive Gag 15mer peptides (data not shown).

These results together indicate that in persons on ART, DC reveal significantly more T cell responses to Gag epitopes than did conventional stimulation with peptide alone.

### Novel HLA B*5101 Gag epitope within p17 revealed by stimulation with peptide-loaded DC

**Figure 6:**
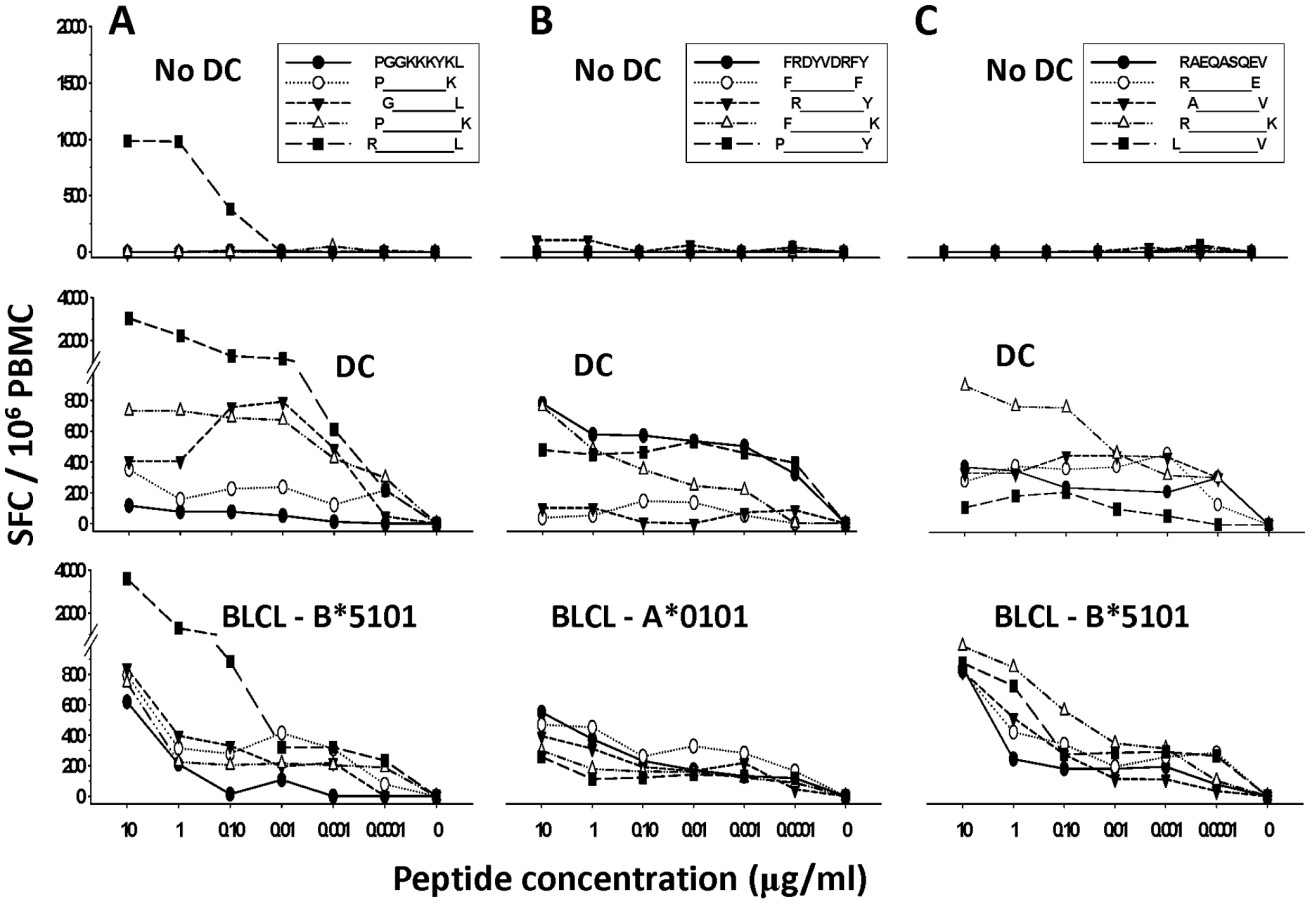
Stimulation of T cells with peptide-loaded DC reveals 3 novel HLA Gag HLA A*0101 and B*5101 epitopes determined by single cell IFN gamma production. Using PBMC from HIV-1 infected subject 2, 3 novel epitopes were identified within Gag 15mer peptides spanning p17_17–35_ and p24_161–183_. Concentration-dependent, single cell IFN gamma production was optimal to peptides with DC for the 10-mer p17_22–31_ (RPGGKKKYKL) for HLA B*5101 (Fig. 6A, DC vs. No DC), 9mer p24_161–169_ (FRDYVDRFY) for HLA A*0101 (Fig. 6B, DC vs. Non DC) and 9mer p24_173–181_ (RAEQASQEV) for HLA B*5101 (Fig. 6C, DC vs. No DC). The HLA restriction of the responses was confirmed by stimulation of the PBMC with peptide-loaded BLCL matched at only one MHC class I allele shown (BLCL, Figs. 6A–C).

We determined whether stimulation of PBMC with peptide-loaded DC revealed novel MHC class I epitopes for Gag. We first focused on Gag p17_17–31_ (EKIRLRPGGKKKYKL) that elicited positive IFNγ responses with DC in 4 of 7 subjects, and its C terminal overlapping peptide p17_21–35_ (LRPGGKKKYKLKHIV), which was positive in 2 of 7 subjects using DC (peptides 5 and 6, Fig. 5, Table S3). A greater IFN-γ response was detected to DC loaded with p17_17–31_ or p17_21–35_ than to peptides without DC in HLA B*5101 positive subject 2 (i.e., 1,585 vs. 9 SFC, and 985 vs. 69 SFC, respectively; P<0.01). Analysis using the HLA Binding Motif Scanner [19] suggested that p17_23–31_ (PGGKKKYKL) was a potential new epitope for HLA B*5101. ELISPOT results for the predicted epitope showed that the 10mer sequence p17_22–31_ (RPGGKKKYKL) stimulated IFN-γ production without DC in a concentration-dependent manner (Fig. 6A, No DC) at levels approximately 2.5-fold higher than without DC. The other peptide variants also induced concentration-dependent IFN-γ production at lower levels than p17_22–31_ when presented by DC (Fig. 6A, DC). We confirmed that the p17_22–31_ 10mer was HLA B*5101-associated by demonstrating optimal, concentration-dependent reactivity using BLCL matched only for this MHC class I allele (Fig. 6A; BLCL- B*5101 matched).

**Figure 7:**
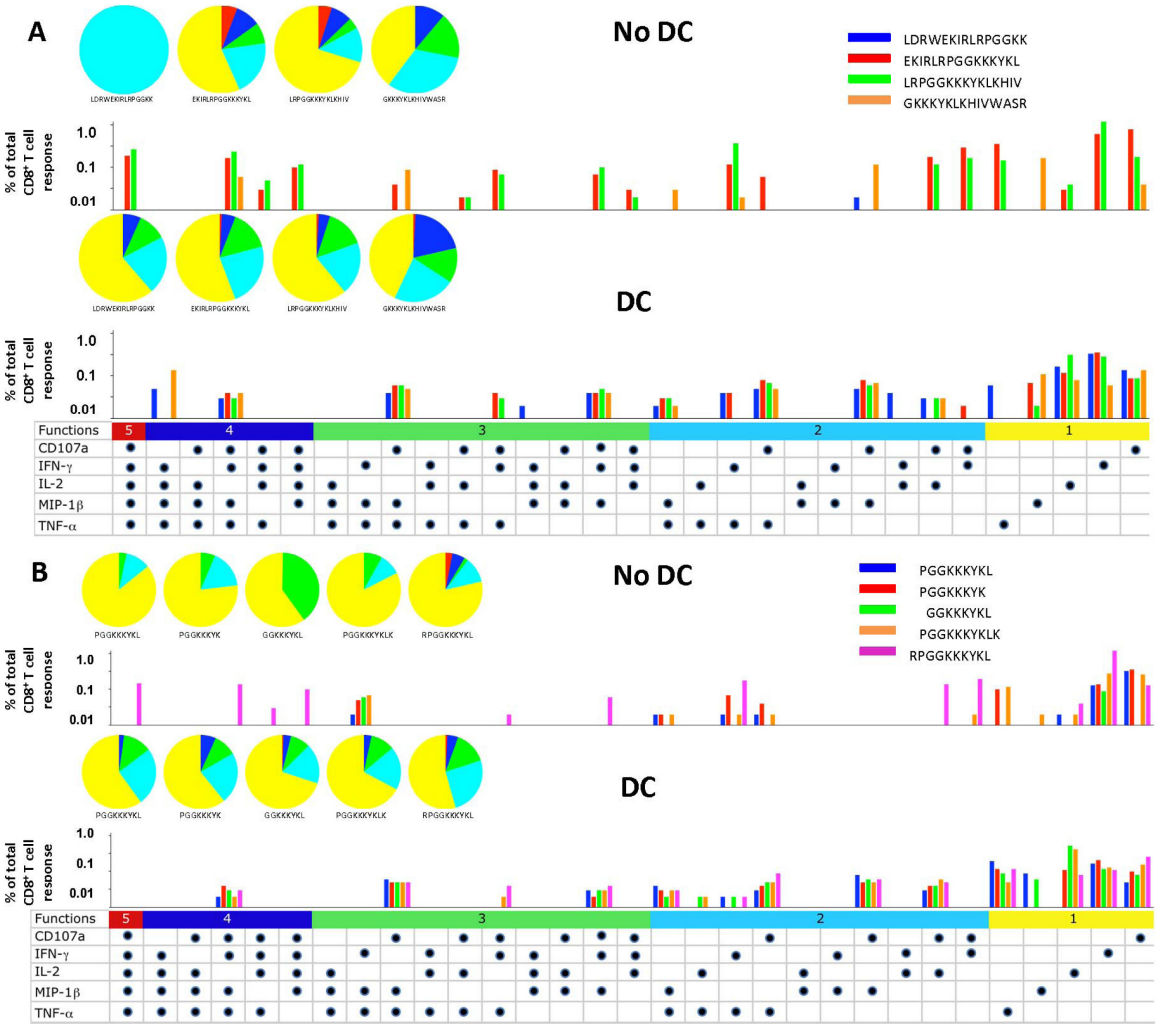
Induction of polyfunctional CD8^+^ T cells by DC loaded with a novel HLA B*5101 Gag p17 epitope. Production of 5 immune mediators by CD8^+^ T cells from HIV-1 infected subject 2 was assessed in response to 4 overlapping 15mer peptides spanning p17_17–35_ (i.e., p17_13–27_, p17_17–31_, p17_21–35_, and p17_25–39_), with and without DC (Fig. 7A), and the in silico predicted, optimal 9mer p17_23–31_ (PGGKKKYKL) for HLA B*5101 and 4 N and C terminal variants with and without DC (Fig. 7B). The details of the graphs are described in Fig. 4.

The overlapping 15mer peptides and the 8-10mer peptides stimulated polyfunctional CD8^+^ T cell reactivity in subject 2 detected by multicolor flow cytometry analysis of CD107a, IFNγ, IL-2, MIP-1β and TNFα. Without DC, higher numbers of monofunctional and polyfunctional CD8^+^ T cells expressed various combinations of these immune mediators in response to the 15mer peptides p17_17–31_ and p17_21–35_ that contained the ELISPOT-optimal p17_22–31_ 10mer, compared to the N and C terminal flanking 15mer peptides p17_13–27_ and p17_25–39_ (P<0.001) (Fig. 7A: no DC). Stimulation with peptide-loaded DC induced monofunctional responses for CD107a, IFNγ and IL-2, and combinations of 2, 3 and 4 polyfunctional responses in CD8^+^ T cells to all 4 of the 15mers (P<0.03 compared to no DC) (Fig. 7A: DC). Of the 8-10mer peptides, in the absence of DC, most monofunctional and polyfunctional responses were induced by the 10mer p17_22–31_, although there was no significant difference compared to the other 4 peptides (P =  ns) (Fig. 7B: no DC). Using peptide-loaded DC, strong monofunctional and polyfunctional CD8^+^ T cell responses of 2-to-4 immune mediators were observed to all 5 8-10mer peptide variants (P = 0.04 compared to no DC) (Fig. 7B: DC).

Thus, using DC and single cell IFN-γ production, we identified Gag p17_22–31_ as a novel, optimal 10mer epitope restricted by HLA B*5101. Polyfunctional CD8^+^ T cell responses induced by the 15mer peptide-loaded DC supported this peptide as the optimal epitope, but were less discriminatory among the 8-10mer, N and C terminal variants presented with or without DC.

### Novel HLA A*0101 and B*5101 Gag epitopes within p24 revealed by stimulation with peptide-loaded DC

A cluster of positive responses resulted from stimulation with peptides 74–76 from the p24 region of Gag, with 4/7 subjects responding to p24_161–175_ (FRDYVDRFYKTLRAE), 5/7 to p24_165–179_ (VDRFYKTLRAEQASQ) and 4/7 to p24_169–183_ (YKTLRAEQASQEVKN), whereas there were no responses to either the N or C terminal flanking 15mers (Fig. 5; Table S3). 12 of 13 positive IFN-γ responses were detected only following stimulation with DC loaded peptides. HLA Binding Motif Scanner [19] predicted that p24_161–169_ (FRDYVDRFY) was a potential new epitope restricted by HLA A*0101, and p24_173–181_ (RAEQASQEV) for HLA B*5101.

Testing of N and C terminal, 8-10mer extensions and truncations of p24_161–169_ showed that there was little or no IFN-γ production induced at any peptide concentration without DC in subject 2 (Fig. 6B, no DC). With DC, however, concentration-dependent IFNγ responses were induced to 3 of these 5 peptides, with the greatest response to the predicted optimal 9mer p24_161–169_ for HLA A*0101 (Fig. 6B, DC). Although no distinct, concentration-dependent reactivity was observed, the greatest responses were induced by the 9mer p24_161–169_ and the 8mer p24_161–168_ (Fig. 6B, BLCL-A*0101 matched).

Concentration-dependent IFNγ ELISPOT responses were maximal with the 10mer p24_173–182_ (RAEQASQEVK), one aa longer than the predicted, optimal HLA B*5101 epitope p24_173–181_ (Fig. 6C, No DC and DC). The response was also greatest to p24_173–182_ presented by BLCL matched only for MHC class I at HLA B*5101 (Fig. 6C, BLCL - B*5101).

**Figure 8:**
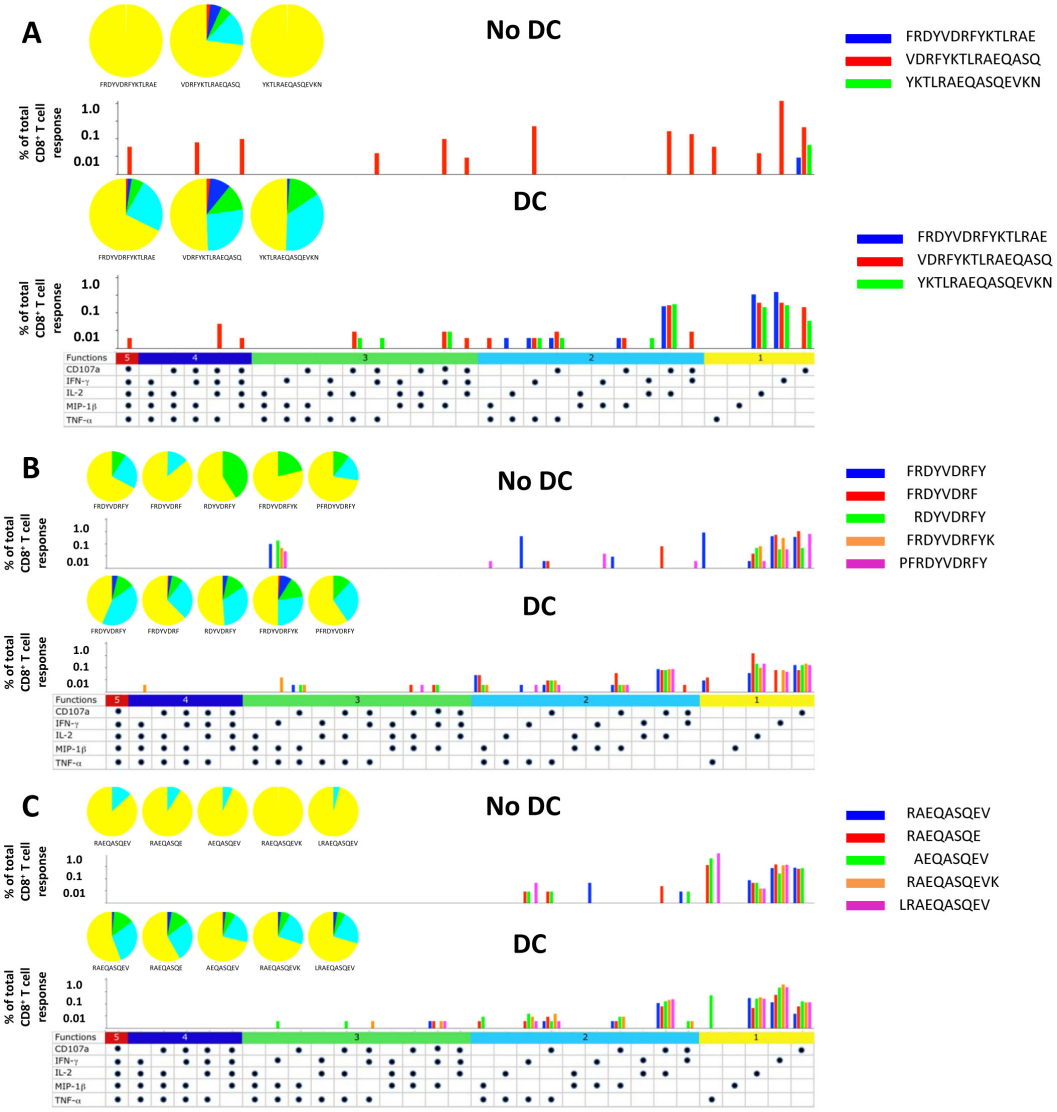
Induction of polyfunctional CD8^+^ T cells by DC loaded with 2 novel HLA A*0101 and B*5101 Gag p24 epitopes. Production of 5 immune mediators by CD8^+^ T cells from HIV-1 infected subject 2 was assessed in response to 3 overlapping 15mer peptides spanning p24_161–183_ (i.e., p24_293–307_, p24_297–311_, and p24_301–315_), with and without DC (Fig. 8A) and the in silico predicted, optimal 9mers and 4 N and C terminal variants of p24_161–169_ (FRDYVDRFY) for HLA A*0101 (Fig. 8B) and p24_173–181_ (RAEQASQEV) for HLA B*5101 (Fig. 8C), with and without DC. The details of the graphs are described in Fig. 4.

Among the 3 overlapping 15mer peptides, optimal monofunctional and polyfunctional CD8^+^ T cell responses were elicited only by p24_165–179_ in the absence of DC (P = 0.02 compared to p24_161–175_ and p24_169–183_) (Fig. 8A, no DC), including cells expressing various combinations of CD107a, IFNγ, IL-2, MIP-1β and TNFα. In contrast, with DC, a broad array of monofunctional and polyfunctional responses were induced in CD8^+^ T cells by p24_165–179_ (P = 0.01 compared to p24_161–175_) and p24_169–183_ (P = 0.07 compared to p24_161–175_) (Fig. 8A, DC).

Without DC, the p24_161–169_ (FRDYVDRFY) peptide and its 8-10mer peptide variants showed no distinct, polyfunctional CD8^+^ T cell responses (P =  ns) (Fig. 8B, no DC). Rather, it induced predominantly monofunctional responses for CD107a, IL-2 and TNFα, and only two polyfunctional responses (IFNγ + MIP-1β + TNFα and IFNγ + TNFα) (P =  ns) (Fig. 8B, no DC). With DC, all 5 peptides induced CD107a + TNFα, CD107a + MIP-1β and CD107a + IL-2 (P<0.02 compared to no DC) (Fig. 8B, DC).

Predominantly monofunctional and some dual polyfunctional CD8^+^ T cell responses were induced by the 5 peptides without DC derived from the putative optimal 9mer p24_173–181_ peptide, with no distinct response to a particular peptide (P =  ns) (Fig. 8C, No DC). Likewise, a broad array of polyfunctional responses to these peptides was detected using DC as APC (P<0.02 compared to No DC) (Fig. 8C, DC). In particular, cells producing CD107a + TNFα and CD107a + IL-2 were induced by all 5 peptides.

Taken together, our data indicate that within this cluster of 3 overlapping Gag p24 15mers, stimulation with peptide-loaded DC revealed a novel HLA A*0101 9mer epitope p24_161–169_, and a novel HLA B*5101 10mer epitope p24_173–182_. N and C terminal variants of these optimal peptides were able to induce appreciable levels of IFN-γ detected by ELISPOT assay only when using DC as APC. There was no clear immunodominance of these 2 Gag p24 epitopes compared to their 8-10mer variants detected by monofunctional or polyfunctional CD8^+^ T cell responses when presented with or without DC.

## Discussion

Control of HIV-1 infection has been related to the magnitude and breadth of HIV-1 CD8^+^ T memory responses, particularly against Gag [1]. While it has been well established that DC are required for priming of naïve CD8^+^ T cells [14], it has only recently been shown that DC are also necessary for optimal activation and expansion of memory CD8^+^ T cells to non-HIV-1 viral infections [14], [15], [16], [17], [18]. We therefore hypothesized that DC could enhance memory T cell reactivity to HIV-1. In support of this hypothesis, we show here that DC loaded with HIV-1 peptides induced the greatest breadth of anti-HIV-1 recall (memory) CD8^+^ T cell reactivity in persons on ART. The IFNγ response induced by peptide loaded DC was mediated by CD8^+^ T cells, with purified CD8^+^ T cells exhibiting an enhanced magnitude and breadth of IFNγ responses relative to PBMC. The 15mer peptides targeted by CD8^+^ T cells were similar to those targeted by PBMC, but included a broader array of peptides across the proteome of HIV-1. CD8^+^ T cell responses were noted to DC loaded with 15mer peptides within all 9 HIV-1 proteins except Vpu. Confirming evidence that CD8^+^ T cells were the predominant responders to HIV-1 peptides was induction of polyfunctional immune mediator reactivity in CD8^+^ T cells to both Gag and Nef peptides.

Focusing on Nef and Gag specificities, we found that T cells responded to clusters of overlapping 15mer Nef and Gag peptides that contained known, immunodominant epitopes matched to the subjects' MHC class I alleles. Notably, T cell reactivity was induced to 22% of these overlapping 49 Nef peptides by DC compared to only 4% of Nef peptides without DC. Furthermore, the magnitude of the anti-Nef responses was significantly greater for peptides with DC compared to peptides without DC. Similar to Nef, positive T cell responses to Gag 15mers containing known MHC class I epitopes were clustered in well documented, immunodominant regions of the protein that matched the MHC class I alleles of our study subjects. DC revealed T cell responses to 16% of these overlapping 122 Gag peptides compared to only 4% of Gag peptides without DC. The magnitude of the anti-Gag responses was also significantly greater for Gag peptides presented by DC compared to peptides without DC.

That autologous, mature DC significantly enhance the breadth and magnitude of CD8^+^ T cell responses to immunodominant epitopes of Nef and Gag has important implications for assessment of T cell immunity in HIV-1 infection. Much of this research relies heavily on use of overlapping 15mer peptides in PBMC without taking into account the role of professional APC [3], [25], [26], [27], [28], [29], [30]. 15mers require processing and presentation by APC, including professional APC, i.e., monocytes, B lymphocytes and myeloid DC, as well as NK and T cells [31], [32], [33], [34], [35], [36]. While it is also possible that this IFN-γ production to 15mers represents MHC class II restricted, CD4^+^ T cell reactivity [37], [38], [39], CD8^+^ T cells were the predominant responders in our study. Our finding implies that the conventional assessment of single cell, IFN-γ responses to libraries of overlapping HIV-1 peptides without DC is potentially missing a significant number of epitopes in persons on ART.

We noted reactivity to 15mer peptides with no previously reported epitope matched for the study subjects' MHC class I alleles for 52% of the reactive Nef peptides and 55% of the reactive Gag peptides. Most of this reactivity was in response to peptides with DC, i.e., T cells specific for 86% of the reactive Nef peptides and 75% of the reactive Gag peptides were only activated by peptide-loaded DC. Analysis of these peptide sequences by predicted binding to their MHC class I motif [19], [20], [21] indicated that many of the peptides contained potential epitopes for the MHC class I alleles of our study subjects. It should be noted that our functional assays could be detecting MHC class I-restricted T cell epitopes on alternative alleles [40]. Furthermore, computational models for predicting MHC-peptide binding are not highly efficient at delineating T cell epitopes [41], [42], [43]. For example, a recent study of influenza A virus [44] showed that only 8% of over 100 viral peptides predicted to bind to MHC class I molecules simulated memory T cell responses in vitro using PBMC from normal adults who presumably had been previously exposed to influenza A virus. Indeed, T cell reactive, viral peptides do not always conform to their putative MHC class I binding motif [45]. It is also notable that DC have not been used as APC in most viral epitope discovery studies [18], [46].

An extensive analysis of a subset of these putative Nef and Gag epitopes indicated that DC indeed revealed novel epitopes for Nef and Gag. Based on single cell IFN-γ production induced by the 15mer Nef_73–87_ with DC, and the predicted binding motif of HLA B*2703, we defined a novel HLA B*2703 epitope, Nef_76–84_ (LRPMTYKAA). This new HLA B*2703 Nef epitope is found in 34% of circulating HIV-1 subtype B sequences, while the most common peptide is LRPMTYKgA found in 42% of circulating sequences (based on a dataset of 1184 subtype sequences). This is a variable peptide region since 77 additional peptides were identified in the 1184 circulating sequences but 73 of them were found in less than 1% of sequences. Similarly, using DC we found 3 novel epitopes within Gag that would have been missed using peptide stimulation alone. The 10mer HLA B*5101 epitope for p17 (p17_22–31_; RPGGKKKYKL) is found in 24% of circulating sequences, while RPGGKKKYrL is found in 27% of sequences. The Gag p24_161–169_ peptide RAEQASQEVK (HLA A*0101 restricted) is the consensus in 49% of circulating HIV-1 sequences, whereas p24_173–182_ FRDYVDRFY (HLA B*5101 restricted) is extremely conserved, found in 99.5% of HIV-1 sequences.

We observed that the 15mer overlapping peptides encompassing the 4 novel Nef and Gag epitopes, as well as the 8-10mer epitope variants within these 15mers, induced monofunctional CD8^+^ T cell reactivity for CD107a, IFNγ, IL-2, MIP-1β, and TNFα, and various polyfunctional combinations of these immune mediators. In particular, the novel Gag peptides presented by DC induced greater levels of polyfunctional CD8^+^ T cells than peptides without DC. However, with or without DC, polyfunctional T cell responses were less discriminatory for optimal epitope specificity than single cell production of IFN-γ by ELISPOT assay. We have recently noted a similar, limited discrimination by polyfunctional analysis of novel T cell epitopes of human herpesvirus 8 presented by DC [47].

There are many factors of mature DC that could relate to their enhancing afferent T cell responses to HIV-1 epitopes. In primary CD8^+^ T cell responses, the majority of immunodominance is based on the affinity of the peptide for its MHC class I allele on APC, forming a stable number of complexes to activate naive T cells [43], [48]. This is also important in stimulation of memory T cells, which are in greater quantity and have a lower threshold for activation than naïve CD8^+^ T cells. However, it is not clear how closely measures of binding of soluble peptides to MHC class I molecules by in vitro affinity assays reflect peptide-MHC binding in DC. Moreover, an increased antigen storage capacity of DC has been linked to their ability to activate T cells by facilitating a continuous supply of MHC class I ligands [49]. DC also enhance the duration of peptide-MHC class I – T cell interactions that are essential for inducing maximum CTL activity [50]. Finally, expression of T cell co-receptors and polarizing, immunomodulatory cytokines such as IL-12 are central to the ability of DC to stimulate antigen-specific CD8^+^ T cells [51], [52], including HIV-1 antigens [53].

Activation of CD8^+^ T cells by DC is also related to the relative expression of T cell receptor and CD8 molecules on memory T cells [54], [55], [56]. The epitope recognition in our study likely involves functional avidity of the antigen-responding T cells, i.e., their capacity to respond to various levels of peptide-MHC class I complexes on the DC [57]. We used a relatively high concentration of peptide with the DC, which could result in preferential stimulation of high avidity, HIV-1 specific T cells [58], as well as cross-reactivity by less avid, non-HIV-1 specific T cells [59]. To limit this effect, we treated the DC with peptide for only 2 h, followed by washing out excess peptide prior to stimulation of the T cells. We also found T cell reactivity to DC loaded with low concentrations of the Nef and Gag peptide epitopes and their variants.

In conclusion, our findings have important implications for T cell immune control of HIV-1 infection. Previously reported, low memory recall, CD8^+^ T cell responses to HIV-1 epitopes in subjects who have suppressed HIV-1 infection on ART may be misleading. Clearly, the present results indicate that mature DC reveal a broad spectrum of T cell epitopes recognized by CD8^+^ T cells in persons on ART with suppressed viral load that are not detectable by conventional stimulation of PBMC with peptide alone. These include novel, MHC class I restricted, HIV-1 epitopes that induce monofunctional and polyfunctional T cells producing up to 5 immune mediators that have been linked to control of HIV-1. This suggests that DC could be potent inducers of anti-HIV-1 T cell immunity as an immunotherapy for HIV-1 infected persons on ART.

## Supporting Information

Table S1 Characteristics of the HIV-1 infected subjects.
(0.21 MB TIF)

Table S2 MHC class I Nef epitopes detected in 7 HIV-1 infected subjects.
(0.03 MB TIF)

Table S3 MHC class I Gag epitopes detected in 7 HIV-1 infected subjects.
(0.03 MB DOCX)
