# Supplementary figures and images for: Dendritic Cells Reveal a Broad Range of MHC Class I Epitopes for HIV-1 in Persons with Suppressed Viral Load on Antiretroviral Therapy
Source: PLoS One. 2010 Sep 23;5(9):e12936. doi: 10.1371/journal.pone.0012936 (PMC2944894; doi:10.1371/journal.pone.0012936)

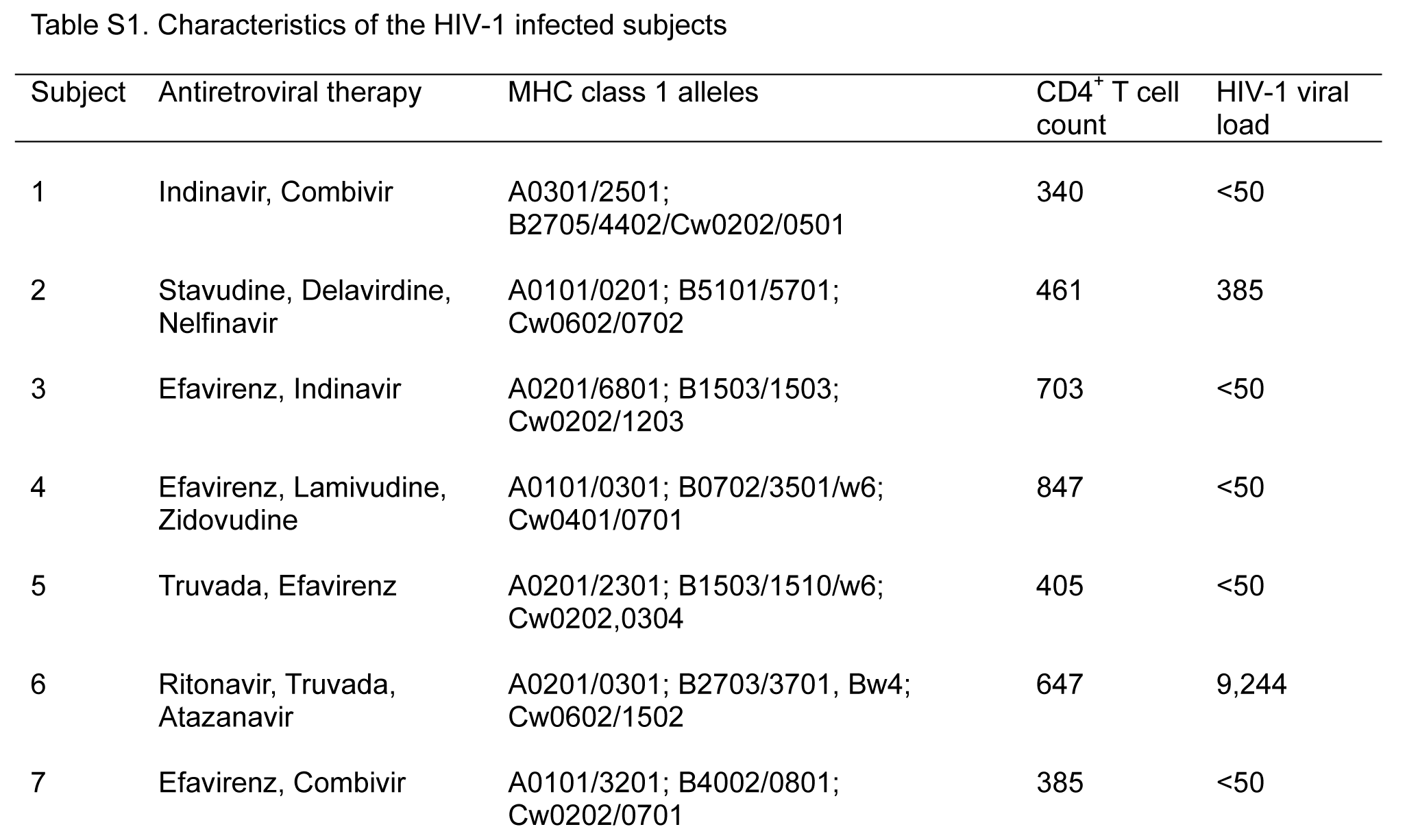

Supplement: Table S1 — Characteristics of the HIV-1 infected subjects. (0.21 MB TIF) [file pone.0012936.s001.tif]
