# Supplementary material for: Dendritic Cells Reveal a Broad Range of MHC Class I Epitopes for HIV-1 in Persons with Suppressed Viral Load on Antiretroviral Therapy
Source: PLoS One. 2010 Sep 23;5(9):e12936. doi: 10.1371/journal.pone.0012936 (PMC2944894; doi:10.1371/journal.pone.0012936)
Supplement: Table S3 — MHC class I Gag epitopes detected in 7 HIV-1 infected subjects. (0.03 MB DOCX) [file pone.0012936.s003.docx]

| **Table S3. HIV-1 Gag MHC class I epitopes detected in 7 HIV-1 infected subjects^a^** | | | | | |
| --- | --- | --- | --- | --- | --- |
|  |  |  |  |  |  |
|  |  |  | Number of IFN Γ responses | Known CD8 epitope^e^ |  |
| Peptide | Location^b^ | Gag peptide (15mer)^c^ | with DC/without DC/both^d^ | (HLA) |  |
| 2 | p17_5-19_ | ASVLSGGELDRWEKI | 1/0/0 | GELDRWEKI (B*4002) |  |
| 3 | p17_9-23_ | SGGELDRWEKIRLRP | 2/0/0 | GELDRWEKI (B*4002) |  |
| 4 | p17_13-27_ | LDRWEKIRLRPGGKK | 2/0/1 | KIRLRPGGK (A*0301; B*27) |  |
|  |  |  |  | IRLRPGGKK (B*2705) |  |
| 5 | p17_17-31_ | EKIRLRPGGKKKYKL | 2/0/2 | KIRLRPGGK (A*0301; B*27) |  |
|  |  |  |  | IRLRPGGKK (B*2705) |  |
|  |  |  |  | RLRPGGKKK (A*0301) |  |
| 6 | p17_21-35_ | LRPGGKKKYKLKHIV | 1/0/1 | LRPGGKKKYKLKHIV (A3) |  |
| 7 | p17_25-39_ | GKKKYKLKHIVWASR | 2/0/1 | None |  |
| 8 | p17_29-43_ | YKLKHIVWASRELER | 2/0/0 | None |  |
| 9 | p17_33-47_ | HIVWASRELERFAVN | 0/0/1 | None |  |
| 10 | p17_37-51_ | ASRELERFAVNPGLL | 1/0/2 | ERFAVNPGL (B27) |  |
| 11 | p17_41-55_ | LERFAVNPGLLETSE | 1/0/2 | ERFAVNPGL (B27) |  |
| 12 | p17_45-59_ | AVNPGLLETSEGCRQ | 2/0/0 | None |  |
| 15 | p17_57-71_ | CRQILGQLQPSLQTG | 2/0/0 | None |  |
| 16 | p17_61-75_ | LGQLQPSLQTGSEEL | 2/0/0 | None |  |
| 17 | p17_65-79_ | QPSLQTGSEELRSLY | 1/0/0 | None |  |
| 19 | p17_73-87_ | EELRSLYNTVATLYC | 5/0/0 | SLYNTVATL (A*0201) |  |
| 20 | p17_77-91_ | SLYNTVATLYCVHQR | 0/0/3 | SLYNTVATL (A*0201) |  |
|  |  |  |  | NTVATLYCV(A2) |  |
| 23 | p17_89-103_ | HQRIEIKDTKEALDK | 1/0/0 | IEIKDTKEAL (B*40) |  |
| 25 | p17_97-111_ | TKEALDKIEEEQNKS | 1/0/0 | None |  |
| 26 | p17_101-115_ | LDKIEEEQNKSKKKA | 2/0/0 | None |  |
| 27 | p17_105-119_ | EEEQNKSKKKAQQAA | 1/0/0 | None |  |
| 28 | p17_109-123_ | NKSKKKAQQAAADTG | 1/0/0 | None |  |
| 29 | p17_113-127_ | KKAQQAAADTGHSNQ | 1/0/0 | None |  |
| 31 | p17_121-132_/p24_1-3_ | DTGHSNQVSQNYPIV | 2/0/0 | None |  |
| 33 | p17_129-132_/p24_1-11_ | SQNYPIVQNIQGQMV | 1/0/0 | None |  |
| 34 | p24_1-15_ | PIVQNIQGQMVHQAI | 1/0/0 | None |  |
| 35 | p24_5-19_ | NIQGQMVHQAISPRT | 1/0/0 | MVHQAISPR (A3 supertype) |  |
| 36 | p24_9-23_ | QMVHQAISPRTLNAW | 3/0/1 | MVHQAISPR (A3 supertype) |  |
|  |  |  |  | HQAISPRTL (B*1501) |  |
|  |  |  |  | QAISPRTLNAW(A*2501) |  |
|  |  |  |  | QAISPRTL (Cw3) |  |
|  |  |  |  | ISPRTLNAW (B*5701) |  |
| 37 | p24_13-27_ | QAISPRTLNAWVKVV | 2/0/0 | QAISPRTLNAW(A*2501) |  |
|  |  |  |  | ISPRTLNAW(B*5701) |  |
|  |  |  |  | TLNAWVKVV (A*0201) |  |
| 40 | p24_25-39_ | KVVEEKAFSPEVIPM | 1/0/0 | None |  |
| 41 | p24_29-43_ | EKAFSPEVIPMFSAL | 0/0/1 | KAFSPEVIPMF (B*5701) |  |
| 42 | p24_33-47_ | SPEVIPMFSALSEGA | 2/0/0 | None |  |
| 45 | p24_45-59_ | EGATPQDLNTMLNTV | 1/0/0 | None |  |
| 48 | p24_57-71_ | NTVGGHQAAMQMLKE | 2/0/0 | GHQAAMQMLKE(A*02) |  |
|  |  |  |  | GHQAAMQML (B*1510) |  |
| 49 | p24_61-75_ | GHQAAMQMLKETINE | 3/0/1 | GHQAAMQMLKE(A*02) |  |
|  |  |  |  | GHQAAMQML (B*1510) |  |
| 52 | p24_73-87_ | INEEAAEWDRVHPVH | 0/0/1 | EEAAEWDRV (B*40) |  |
|  |  |  |  | AEWDRVHPV (B*4002) |  |
| 53 | p24_77-91_ | AAEWDRVHPVHAGPI | 1/0/0 | HPVHAGPI (B35) |  |
| 54 | p24_81-95_ | DRVHPVHAGPIAPGQ | 2/0/0 | HPVHAGPIA (B7; B35) |  |
| 56 | p24_89-103_ | GPIAPGQMREPRGSD | 1/0/0 | None |  |
| 57 | p24_93-107_ | PGQMREPRGSDIAGT | 1/0/0 | None |  |
| 63 | p24_117-131_ | WMTNNPPIPVGEIYK | 0/1/0 | MTNNPPIPV (A*0201) |  |
| 64 | p24_121-135_ | NPPIPVGEIYKRWII | 0/1/1 | GEIYKRWII (B*0801) |  |
| 65 | p24_125-139_ | PVGEIYKRWIILGLN | 1/1/2 | GEIYKRWII (B*0801) |  |
|  |  |  |  | EIYKRWII (A2) |  |
|  |  |  |  | IYKRWIILGL(B27) |  |
| 66 | p24_129-143_ | IYKRWIILGLNKIVR | 2/2/1 | IYKRWIILGL (B27) |  |
|  |  |  |  | RWIILGLNK (B27) |  |
|  |  |  |  | IILGLNKIV (A2) |  |
|  |  |  |  | IILGLNKIVR (A3) |  |
| 67 | p24_133-147_ | WIILGLNKIVRMYSP | 1/0/0 | IILGLNKIVR (A3) |  |
|  |  |  |  | ILGLNKIV (B*27) |  |
| 68 | p24_137-151_ | GLNKIVRMYSPTSIL | 0/0/1 | RMYSPTSIL (A2) |  |
| 69 | p24_141-155_ | IVRMYSPTSILDIRQ | 1/0/0 | RMYSPTSIL (A2) |  |
| 74 | p24_161-175_ | FRDYVDRFYKTLRAE | 3/1/0 | FRDYVDRFYK (B27) |  |
|  |  |  |  | RDYVDRFYKTL (B*4402) |  |
|  |  |  |  | YVDRFYKTL (A*02) |  |
| 75 | p24_165-179_ | VDRFYKTLRAEQASQ | 5/0/0 | VDRFYKTLRAEQASQ (B*57) |  |
| 76 | p24_169-183_ | YKTLRAEQASQEVKN | 4/0/0 | None |  |
| 80 | p24_185-199_ | MTETLLVQNANPDCK | 1/0/0 | None |  |
| 82 | p24_193-207_ | NANPDCKTILKALGP | 1/0/0 | None |  |
| 83 | p24_197-211_ | DCKTILKALGPAATL | 1/0/0 | None |  |
| 84 | p24_201-215_ | ILKALGPAATLEEMM | 2/0/0 | None |  |
| 85 | p24_205-219_ | LGPAATLEEMMTACQ | 1/0/1 | ATLEEMMTA (A2) |  |
| 86 | p24_209-223_ | ATLEEMMTACQGVGG | 3/0/1 | EMMTACQGV (A*0201) |  |
| 87 | p24_213-227_ | EMMTACQGVGGPGHK | 2/0/0 | EMMTACQGV (A*0201) |  |
| 92 | p2_2-14_;p7_1-2_ | EAMSQVTNSATIMMQ | 1/1/0 | SQVTNSATI (A*02) |  |
| 93 | p2_6-14_;p7_1-6_ | QVTNSATIMMQRGNF | 0/1/0 | None |  |
| 94 | p2_10-14_;p7_1-10_ | SATIMMQRGNFRNQR | 1/0/0 | None |  |
| 95 | p2_14_;p7_1-14_ | MMQRGNFRNQRKIVK | 1/1/0 | None |  |
| 96 | p7_4-18_ | GNFRNQRKIVKCFNC | 3/0/0 | None |  |
| 97 | p7_8-22_ | NQRKIVKCFNCGKEG | 1/0/0 | None |  |
| 98 | p7_12-26_ | IVKCFNCGKEGHTAR | 1/0/0 | None |  |
| 99 | p7_16-30_ | FNCGKEGHTARNCRA | 1/0/0 | None |  |
| 102 | p7_28-42_ | CRAPRKKGCWKCGKE | 2/1/0 | None |  |
| 103 | p7_32-46_ | RKKGCWKCGKEGHQM | 2/0/0 | None |  |
| 104 | p7_36-50_ | CWKCGKEGHQMKDCT | 2/0/0 | None |  |
| 105 | p7_40-54_ | GKEGHQMKDCTERQA | 1/1/0 | None |  |
| 106 | p7_44-55_;p1_1-3_ | HQMKDCTERQANFLG | 2/0/0 | TERQANFL (B*4002) |  |
| 107 | p7_48-55_;p1_1-7_ | DCTERQANFLGKIWP | 1/0/0 | None |  |
| 108 | p7_52-55_;p1_1-11_ | RQANFLGKIWPSYKG | 0/0/1 | FLGKIWPSYK (A*0201) |  |
| 109 | p1_1-15_ | FLGKIWPSYKGRPGN | 0/0/1 | FLGKIWPSYK (A*0201) |  |
| 111 | p1_9-16_;p6_1-7_ | YKGRPGNFLQSRPEP | 1/0/0 | None |  |
| 114 | p6_5-19_ | PEPTAPPEESFRSGV | 1/0/1 | None |  |
| 115 | p6_9-23_ | APPEESFRSGVETTT | 1/0/0 | None |  |
| 116 | p6_13-27_ | ESFRSGVETTTPPQK | 2/0/0 | None |  |
| 117 | p6_17-31_ | SGVETTTPPQKQEPI | 1/0/0 | None |  |
| 118 | p6_21-35_ | TTTPPQKQEPIDKEL | 0/1/0 | None |  |
| 121 | p6_33-47_ | KELYPLTSLRSLFGN | 2/0/0 | YPLTSLRSLF (B7) |  |
| 122 | p6_37-52_ | PLTSLRSLFGNDPSSQ | 2/0/0 | None |  |
| ^a^ Known epitopes based on the CTL/CD8^+^ T-cell Epitope Database Epitope Location Finder (hiv-web.lanl.gov). | | | | |  |
| ^b^ Results shown are positive responses to peptides derived from HIV-1 Gag HXB2 Clade B. | | | | |  |
| ^c^ HIV-1 15mer peptides overlapping by 11 amino acids and spanning HIV-1 Gag. | | | |  |  |
| ^d^ # positive IFNγ responses with DC/without DC/both with DC and without DC, in total of 7 subjects. | | | | |  |
| ^e^ Minimal sequences for known epitopes with an associated HLA haplotype. | | | |  |  |
